# Lower Extremity Salvage with Thoracodorsal Artery Perforator Free Flap in Condition of Symmetrical Peripheral Gangrene

**DOI:** 10.1155/2018/6508607

**Published:** 2018-05-08

**Authors:** Soo Yeon Lim, Gyeong Hoe Kim, Il Hoon Sung, Dong Woo Jang, Jung Soo Yoon, Youn Hwan Kim, Sang Wha Kim

**Affiliations:** ^1^Department of Plastic and Reconstructive Surgery, College of Medicine, Hanyang University, Seoul, Republic of Korea; ^2^Department of Plastic and Reconstructive Surgery, College of Medicine, Seoul National University, Seoul National University Hospital, Seoul, Republic of Korea; ^3^Department of Orthopaedic Surgery, College of Medicine, Hanyang University, Seoul, Republic of Korea; ^4^Shanghai MK Medical Cosmetic Surgery Clinic, Shanghai, China

## Abstract

Symmetrical peripheral gangrene (SPG) is rare but devastating complication which is characterized by symmetrical ischemic change of the distal extremities. In this report, we describe our management protocol for SPG, focusing on surgical approaches. Between January 2007 and February 2016, 10 thoracodorsal artery perforator (TDAP) free flaps were performed in 6 patients with SPG. Three patients were male and mean age was 56 (range, 44–69) years. All the patients were in shock. The causes of shock were sepsis in 4 cases, respiratory arrest in 1 case, and hypovolemia in 1 case. Eight transmetatarsal amputations and 2 Lisfranc amputations were performed. Flap sizes ranged from 7 × 11 cm to 25 × 15 cm. There were 3 cases of partial necrosis of the flap: two healed conservatively with dressings and one required skin graft. Three of the patients were later able to walk independently at Functional Ambulation Classification (FAC) level 6, one patient could walk independently on level surfaces at FAC level 5, and 2 could walk independently using walking aids, classified at FAC level 4. The average follow-up period was 18 (range, 6–54) months. In patients with SPG, minimal bone amputation and foot salvage with TDAP flaps were successful. Separate reconstruction of bone and soft tissue had good outcomes.

## 1. Introduction

Symmetrical peripheral gangrene (SPG) is a rare but devastating complication characterized by symmetrical ischemic change of the distal extremities. Mortality is over 40%, and about half of the patients who survive require amputation [1]. The cause of SPG is not well understood, and various infective and noninfective factors have been suggested. Disseminated intravascular coagulation (DIC) is present in 85% to 100% cases [1–3] and it is generally due to sepsis [2, 3]. Shock itself can also contribute to the occurrence of SPG by reducing cardiac output and causing reflex peripheral vasoconstriction. Moreover, vasopressor agents, which are commonly used in the management of sepsis-induced hypotension, are aggravating factors [1, 4, 5].

SPG can progress to gangrene of two or more sites without large vessel obstruction [1, 4, 6]. The ischemic change begins distally and may progress proximally to involve the entire extremity. It is difficult to prevent the development of gangrene, and therefore amputation is inevitable. It may be inevitable but it is rarely urgent. Initially a nonsurgical approach to management is preferred to allow time for improvement of the medical condition of the patient and to allow necrotic areas to become demarcated [7–13]. Afterwards, appropriate surgical procedures, including reconstructive procedures, are required to salvage the extremities. Salvage of the length of the extremities is extremely important to preserve their function, especially in the case of lower limbs. Reconstruction with free flaps can be particularly effective in this regard [14, 15].

In this report, we describe our management protocol for SPG, focusing on surgical approaches. Forefoot or midfoot amputation is performed first, and then skin and soft tissue are reconstructed with thoracodorsal artery perforator (TDAP) free flaps, which are an alternative option for SPG in terms of wound healing and functional outcome. To the best of our knowledge, this is first consecutive series using microsurgical tissue transfer for reconstructing foot defects in SPG.

## 2. Patients and Methods

Between January 2007 and February 2016, 10 TDAP free flaps were performed in 6 patients with SPG. Three patients were male and three were female. All were in shock accompanied by DIC due to a variety of causes and had SPG.

Mean age was 56 (range, 44–69) years. Five patients had hypertension and diabetes mellitus, one of whom received peritoneal dialysis due to end-stage renal disease. The causes of shock were sepsis in 4 cases, respiratory arrest in 1 case, and hypovolemia due to aortic dissection in the remaining case. For the treatment of sepsis, 2 patients were treated with injections of epinephrine and the other 2 with norepinephrine; the remaining 2 patients received dopamine. Five of the patients had SPG on both feet, 2 of whom had it on both hands as well. The sixth patient had unilateral gangrene on the right foot. Detailed information on each patient was collected from the medical records (Table 1).

### 2.1. Surgical Procedure

Patients stayed in the intensive care unit while in shock. After recovery, they were transferred to general wards. When their general condition had stabilized and necrotic areas had been demarcated, radical debridement of necrotic tissue was performed. At the same time, amputation was performed by an orthopedic surgeon. The amputation level was determined on the basis of the radiographic and gross intraoperative findings. After debridement and amputation, negative pressure wound therapy (V.A.C. system, KCI Corporation, San Antonio, TX, USA) was applied. The patients underwent serial debridement every 24–72 hours to ensure that the wounds were stabilized and healthy granulation tissue wound grew. Afterwards, reconstructive surgery was considered. All the patients underwent computed tomography (CT) angiography to confirm vascularity of the lower extremities and to locate reliable recipient vessels.

All the defects were reconstructed using TDAP free flaps. To harvest the flaps, the patient was placed in a supine position with the arm abducted and elevated. An incision was made along the midline between the border of the pectoralis major and latissimus dorsi (LD) muscle. To avoid missing very small perforators, traction and counter-traction were performed. One or two reliable (i.e., “pulsatile”) perforators were located along the anterior border of the LD muscle. If no perforators penetrating the LD muscle were found, septocutaneous or direct cutaneous perforators were located instead. The branch of the thoracodorsal artery perforator pedicle that entered the perforators to the skin paddles was dissected. If muscle was required, other branches of the thoracodorsal artery, such as transverse branches feeding the LD muscle, could be harvested with LD muscle flaps in a chimeric pattern. After identifying suitable vessels for the flap, an outline of the flap appropriate for the defect was made. The flap was then harvested in cephalic direction. We ensured that the pedicle length exceeded 15 cm, which was sufficient for any location of the recipient vessels. If a thin flap was required, the flap included only the superficial subcutaneous fat layers, and the deep fat layers were discarded.

After insetting the flap into the defect, microanastomosis was performed. Microanastomosis of the artery was achieved in an end-to-side fashion using 9-0 suturing materials. The venous return was checked. Vein anastomosis proceeded in an end-to-end fashion. The donor site was closed primarily or a split thickness skin graft was applied. Prostaglandin was administrated intravenously for 2 weeks postoperatively.

## 3. Results

A total of 10 cases of foot amputation and reconstruction with TDAP free flaps were performed in 6 patients. For hand gangrene, only amputation and skin closure were performed after demarcation. One patient with both feet gangrene underwent simple amputation of the left foot. Forefoot or midfoot amputations were performed in all cases; these consisted of 8 transmetatarsal amputations and 2 Lisfranc amputations.

Flap size varied from 7 × 11 cm to 25 × 15 cm. The mean pedicle length of the flaps was 8.5 cm, ranging from 7 cm to 11 cm. All TDAP flaps included one perforator except for one chimeric flap including a 4 × 4 cm LD muscle flap. The TDAP flaps were used to resurface the whole foot defects, and split thickness skin grafts were performed in 2 cases to cover the defects not covered by the TDAP free flaps. The recipient vessels used were located above the ankle level away from the zones of injury. The anterior tibial vessels were used in 5 cases and posterior tibial vessels in the 5 others. After harvesting the flaps, the donor sites were closed primarily in 6 cases, and split thickness skin grafts were performed in the 4 other cases. All the donor sites healed well without any complications.

In 3 cases, partial necrosis of the flap occurred. Although two partial necrosis cases healed conservatively with dressings, one required an additional split thickness skin graft.

The latter patient was discharged after five months. All the other patients were discharged within 3 months after total wound healing and began rehabilitation. Final ambulation states and late complications were evaluated in outpatient clinics during the follow-up period. Ambulatory states were evaluated by the Functional Ambulation Classification (FAC) in outpatient clinics (Table 2) [16]. All the patients were able to walk alone. Three were able to walk independently at FAC level 6 using foot orthoses and foot-wear, and 1 could walk independently on level surfaces at FAC level 5. The remaining 2 patients could walk independently at FAC level 4 using walking aids. The average FAC level was 5.2.

During the follow-up period, chronic ulceration occurred in one patient after weight-bearing because of high-pressure on a bony prominence. Additional ostectomy and wound closure were performed under local anesthesia. Another patient had chronic osteomyelitis after transmetatarsal amputation, and further amputation was performed at the metatarsal level.

The average follow-up period was 18 months, ranging from 6 to 54 months.

## 4. Case Reports

### 4.1. Case 1

**Figure 1:**
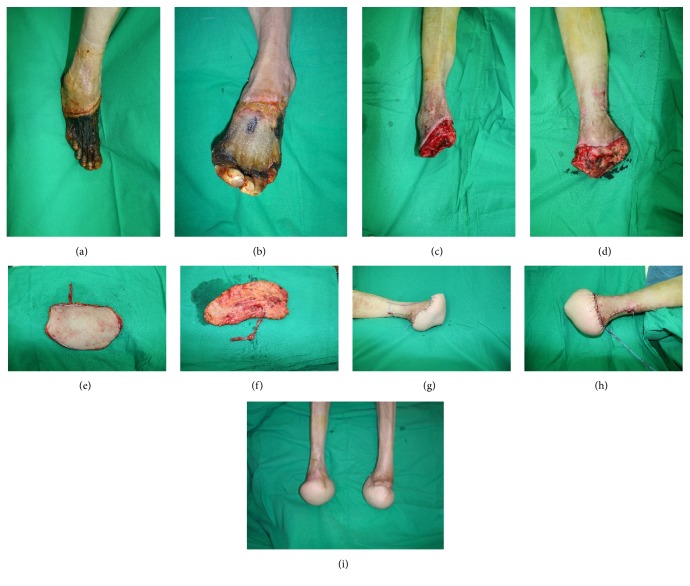
(a) and (b) Necrosis of both ischemic feet after use of norphin in septic shock: (a) Rt. Foot and (b) Lt. Foot. (c) Lisfranc amputation and debridement of skin and soft tissue were performed. (d) Transmetatarsal amputation and debridement of skin and soft tissue were performed. (e) A thoracodorsal artery perforator flap was harvested for Rt. Foot reconstruction. (f) A thoracodorsal artery perforator flap was harvested for Lt. Foot reconstruction. (g) Immediate postoperative view of Rt. Foot. (h) Immediate postoperative view of Lt. Foot. (i) Postoperative 6-month view.

A 60-year-old female patient with a history of diabetes-induced end-stage renal disease presented with septic shock. After two months managing the septic shock with norepinephrine, her general condition improved, but both feet were necrotic (Figures 1(a) and 1(b)). First, bone amputation and debridement of skin and soft tissue were performed. A Lisfranc amputation was performed on the right foot (Figure 1(c)) and a transmetatarsal amputation on the left foot (Figure 1(d)). Afterwards, two more serial debridements of soft tissue were carried out until the wound was ready for reconstruction. A chimeric TDAP flap (9 × 12 cm) with a latissimus dorsi muscle flap (4 × 4 cm) was planned to cover the defect in the right foot (Figure 1(e)) and cushion the remaining heel, and a 20 × 12 cm TDAP flap was planned for the left foot (Figure 1(f)). The posterior tibial artery was used for the anastomosis in the right foot and the anterior tibial artery in the left foot, both in end-to-end manner (Figures 1(g) and 1(h)). As the TDAP flap for the left foot was large, the donor site required skin grafting, while the donor site for the right foot was closed primarily. The partial necrosis of the right TDAP flap healed without surgery. During 12 months of follow-up, the patient could walk using a walking aid and was classified at FAC level 4 (Figure 1(i)).

### 4.2. Case 2

**Figure 2:**
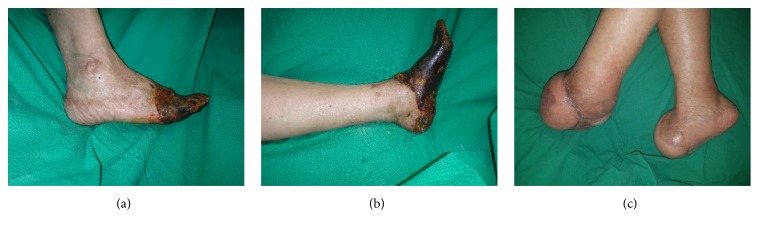
(a) and (b) Necrosis of both ischemic feet after use of norphin in septic shock: (a) Rt. Foot and (b) Lt. Foot. (c) Postoperative 6-month view. Both necrotic feet underwent midfoot amputation and reconstruction with TDAP free flaps.

A 69-year-old female patient with a history of hypertension and diabetes presented with SPG of both feet after management of septic shock with norepinephrine (Figures 2(a) and 2(b)). The orthopedic surgeon performed transmetatarsal amputation of both feet, and the plastic surgeon debrided necrotic tissue. After 3 serial debridements, both feet were reconstructed with 15 × 10 cm TDAP flaps anastomosed to the anterior tibial artery in the right foot and a 25 × 15 cm TDAP flap anastomosed to the posterior tibial artery in the left foot. The right TDAP donor site was closed primarily but the left flap required a skin graft. The left flap was partially necrotic and healed conservatively. However, 3 months after reconstructing the left foot, chronic ulceration was found due to chronic osteomyelitis. Further metatarsal amputation was performed at the metatarsal level, and the wound was closed. Despite the additional operation, the patient was able to walk (Figure 2(c)).

## 5. Discussion

Symmetrical peripheral gangrene (SPG) is a devastating condition characterized by ischemic gangrene affecting 2 or 4 limbs symmetrically and may require amputation of the affected limbs. Such gangrene results from peripheral vasoconstriction and decreased cardiac output [9–13]. SPG has risk factors such as peripheral vascular disease, sepsis, DIC, diabetes mellitus, acute kidney injury, and obesity. Shock itself can contribute to the occurrence of SPG by reducing cardiac output and causing reflex peripheral vasoconstriction. DIC, especially, can cause the occlusion of peripheral vessels in septic shock, increasing the extent of SPG in peripheral extremities in conjunction with the use of vasopressors [13, 17, 18]. Dopamine or noradrenaline is commonly used as a first-line vasopressor to manage low pressure in shock patients. Since Holzer et al. reported in 1973 that prolonged high doses of dopamine and noradrenaline administration can cause peripheral gangrene [9, 10], the use of vasopressors is known to be one of the factors contributing to peripheral gangrene via vasoconstriction of peripheral vessels.

There are no specific preventions or treatments for SPG. As a result, amputation is often inevitable due to the irreversibly necrotic tissue that can cause secondary infection [14]. In the past decades, there have been efforts to preserve the length of the lower extremity by more distal amputation [19]. The amputation level chosen should be based on having sufficient soft tissue coverage for effective wound healing and preserving as much of the length of the limb as possible for functional and aesthetic reasons. The appropriate level of amputation depends on several factors. First, radical debridement of the necrotic tissue of the ischemic foot gangrene is needed because remnant necrotic tissue can cause infection. In addition, sufficient skin and soft tissue is required for wound closure and cushioning the effect on the weight-bearing surface. The vascularity of the remnant tissue should also be taken into account to permit wound healing. In addition, due to the characteristics of the lower extremity, especially the foot, a balanced procedure for amputation is often required to help with weight-bearing and walking [19–21].

Deciding the amputation level by evaluating peripheral vascularity is essential before surgery. The decision is based on physical findings, such as color, temperature, and peripheral pulse, and imaging studies, such as Doppler sonography and CT angiography. Detecting bone involvement from imaging studies, such as X-ray, bone scan, and MRI, can be useful. Intraoperative findings like wound bleeding or color changes should be taken into consideration because bone is not necessarily involved when there is skin and soft tissue necrosis [19, 20]. In our cases, amputation was performed simultaneously with radical debridement of necrotic tissue.

Bony amputation was performed by an orthopedic surgeon, and the level of amputation was determined only by the involvement of bone. After bony amputation, necrotic skin and soft tissue was serially debrided, and a negative pressure dressing was applied for two-three days between debridement procedures until the wound was clear and ready for reconstruction. Bone and skin and soft tissue were considered separately, and amputation of the bone was performed first, followed by soft tissue reconstruction. Therefore, the volume of the remaining skin and soft tissue and the requirements of wound healing had less influence on the choice of amputation level. The procedure for reconstruction of skin and soft tissue, which usually required free flaps, was only decided after sufficient debridement. As skin and soft tissue necrosis was usually more extensive than bone necrosis, the amputation level needed to be more proximal to be able to cover the amputated bone ends with remaining skin and soft tissue. Therefore, if bone and soft tissue are separately taken into account, this can lower the level of amputation and preserve the bony length of the foot.

Only two cases required Lisfranc amputation, the others undergoing transmetatarsal amputation (TMA). In ischemic foot gangrene, TMA can save important structures, such as the insertions of the tibialis anterior, peroneus longus, and brevis tendons, and preserve the important functioning of the ankle joint [20]. Furthermore, ambulatory function can be maintained by preserving the sensate heal [22–24]. However, the major drawback of TMA is impaired wound healing due to insufficient vascularity and lack of sufficient skin and soft tissue [22, 24–27]. Many workers report that the wound healing rate is only 40%–70%, and more proximal amputation should be considered in some cases [22, 25–27]. More proximal Lisfranc amputation can provide more adequate skin and soft tissue and improve the wound healing process, but it sacrifices important tendon and foot structures and leads to major muscle imbalance and functional deformity. If equinus deformity occurs, the weight-bearing balance of the foot is destroyed, which may lead to chronic ulceration and cause ambulation dysfunction [20, 21]. An additional orthopedic operation, such as Achilles tendon lengthening and posterior capsulotomy of the ankle or subtalar joint, is then necessary to overcome this deformity [20, 21, 23], making TMA a more favorable option [20, 23]. On the other hand, if reconstruction of skin and soft tissue to cover the defect is not successful, or wound healing complications occur in TMA or Lisfranc amputations, more proximal amputation, below-knee amputation, should be considered [19–21].

After amputation and radical debridement, reconstruction of skin and soft tissue defects and coverage of exposed bone has to be considered. To resurface large and complex defects with accompanying bone exposure, overcome wound healing complications, and maximize functional and aesthetic outcomes, foot reconstruction is usually performed by transfer of vascularized free tissue [28–31]. Recently, several articles have advocated the use of thoracodorsal artery perforator (TDAP) free flaps for foot reconstruction [28–32]: a large and pliable TDAP flap can be elevated with less donor site morbidity [32], and long vascular pedicles up to 18 cm can be taken, permitting the use for anastomosis of large, reliable proximal vessels outside of the zone of injury [28–31]. Moreover, the incidence of atherosclerotic change in thoracodorsal arteries is low compared to the lower limb, making the pedicles reliable. Perforator flaps are pliable and super-thin—under 5 mm thick—making them useful for foot resurfacing and subsequent wearing of shoes. TDAP flaps include thick skin tissue from the flank and back region, which helps with weight-bearing and provides the plantar structure with resistance to friction [28–32]. Besides that, chimeric patterned flaps provide versatile options for reconstructing complex foot defects. Muscle flaps with sufficient blood supply help to obliterate dead space and control subclinical infection. The donor site can be closed primarily, and a two-team approach is possible [28–32].

Minimal bony amputation and foot reconstruction with TDAP flaps provide successful foot salvage in SPG. However, even if reconstruction is successful, for ambulation to be possible, active postoperative rehabilitation is essential, and patients need to be strong-willed to obtain good results [19–21]. In most of our cases the patients healed well without major wound healing problems, and a mean ambulation function of FAC level 5.2 was achieved. The patients were satisfied with the functional and aesthetic results.

## 6. Conclusions

To manage SPG, forefoot and midfoot amputation was performed first, and then the foot was reconstructed with TDAP free flap. We should bear in mind that since necrosis of skin and necrosis of bone are different, the amputation level should be decided intraoperatively. Moreover, separate reconstruction of bone and soft tissue leads to good outcomes in terms of both wound healing and ambulatory status.

## Figures and Tables

**Table 1:** Summary of patient characteristics.

Gender/age	Shock state	Vasopressor	Underlying disease	Involved region	Amputation level	Type of flap	Flap size (cm)	Pedicle length (cm)	Recipient vessels	Donor site	Early complications	Late complications	Ambulation (FAC)	Follow-up (month)
M/44	Septic shock	Epinephrine	HTN, DM	Feet, both	Transmetatarsal amputation, Rt.	TDAP	7 × 11	7	ATA, AV	Primary closure	None		6	18

M/46	Respiratory arrest	Epinephrine	HTN, DM	Feet, both hands, both	Transmetatarsal amputation, Rt.	TDAP	8 × 10	10	PTA, AV	Primary closure	None		6	12
					Transmetatarsal amputation, Lt.	TDAP, STSG	9 × 11	10	PTA, AV	Primary closure	None			

M/60	Hypovolemic shock	Dopamine	Aortic dissection (Aorto-bi-iliac bypass graft state)	Foot, Rt.	Lisfranc amputation, Rt.	TDAP	8 × 12	8	ATA, AV	Primary closure	None	Chronic ulcer	5	54

F/60	Septic shock	Norphin	HTN, DM, ESRD, CHF, PTE	Feet, both	Lisfranc amputation, Rt.	TDAP & LDm	9 × 12	7	ATA, AV	Primary closure	Partial necrosis		4	12
						(Chimeric)	(4 × 4)							
					Transmetatarsal amputation, Lt.	TDAP	20 × 12	7	PTA, GSV	STSG coverage	None			

F/57	Septic shock	Dopamine	HTN, DM	Feet, both hands, both	Transmetatarsal amputation, Rt.	TDAP	20 × 15	7	PTA, AV	STSG coverage	Partial necrosis, STSG		4	6
					Transmetatarsal amputation, Lt.	TDAP	25 × 15	11	ATA, AV	STSG coverage	None			

F/69	Septic shock	Norphin	HTN, DM	Feet, both	Transmetatarsal amputation, Rt.	TDAP	15 × 10	7	ATA, AV	Primary closure	None	Chronic	5	6
					Transmetatarsal amputation, Lt.	TDAP, STSG	25 × 15	11	PTA, AV	STSG coverage	Partial necrosis	Osteomyelitis		

HTN: hypertension; DM: diabetes mellitus; ESRD: end-stage renal disease; CHF: congestive heart failure; PTE: pulmonary thromboembolism; TDAP: thoracodorsal artery perforator; ATA: anterior tibial artery; PTA: posterior tibial artery; AV: accompanying vein; GSV: greater saphenous vein; STSG: split thickness skin graft; OM: osteomyelitis.

**Table 2:** Functional Ambulation Classification (FAC) assessment scale levels.

FAC level	Ambulation description	Definition
1	Nonfunctional	Unable to ambulate

2	Dependent, level II	Requires manual contact of one person during ambulation on level surfaces Manual contact is continuous and necessary to support body weight and/or to maintain balance or assist coordination

3	Dependent, level II	Requires manual contact of one person during ambulation on level surfaces Manual contact is continuous or intermittent light touch to assist balance or coordination

4	Dependent, supervision	Ambulation occurs on level surfaces without manual contact of another person Requires stand-by guarding of one person because of poor judgement, questionable cardiac status, or the need for verbal cuing to complete the task

5	Independent, level surfaces only	Ambulation is independent on level surfaces Requires supervision/physical assistance to negotiate stairs, inclines, or unlevel surfaces

6	Independent, level and nonlevel surfaces	Ambulation is independent on unlevel and level surfaces, stairs, and inclines
